# Virtual front door for new referrals from a Diabetic Eye Screening Programme in England

**DOI:** 10.1038/s41433-023-02865-6

**Published:** 2023-11-30

**Authors:** Heather M. Wood, Sarita Jacob

**Affiliations:** 1 University Hospitals Birmingham NHS Trust, Birmingham, England; 2 College of Medical and Dental Sciences, University of Birmingham, Birmingham, England; 3 Birmingham, Solihull and Black Country Diabetic Eye Screening Programme, Birmingham, England; 4 College of Health and Life Sciences, Aston University, Birmingham, England

**Keywords:** Health services, Education

## Introduction

Diabetic retinopathy (DR) is a leading cause of visual impairment in the UK [1]. Early detection of DR through national diabetic eye screening programme[s] (DESP) has been shown to preserve vision and reduce the necessity for late treatment [2]. Virtual clinics for follow-up DR patients have been increasingly utilised in recent years following the Covid-19 pandemic. We collected evidence relating to compliance with national guidelines relating to waiting times for *routine new diabetic referrals*, time frame for follow-ups, mean time-to-treatment, and distribution of subsequent face-to-face and virtual follow-up for a new virtual Diabetic Retinopathy Imaging Clinic (DRIC).

## Methods

We audited the performance of the virtual DRIC at University Hospitals Birmingham NHS Trust purely for new referrals from the DESP between May 2021 and 2022 which utilised a technician-led diagnostic hub (Full history, vision, IOP, ultra-widefield imaging and OCT) followed by next day consultant review. The patient cohort deemed to be suitable for the DRIC included patients with non-proliferative diabetic retinopathy (DR), maculopathy, and those with non-DR referrals from DESP (including choroidal naevi, wet AMD, and RVO). Data was recorded relating to patient demographics, referral DR grade, DRIC DR grade, non-DR lesions, outcomes, and follow-up time.

## Results

**Fig. 1:**
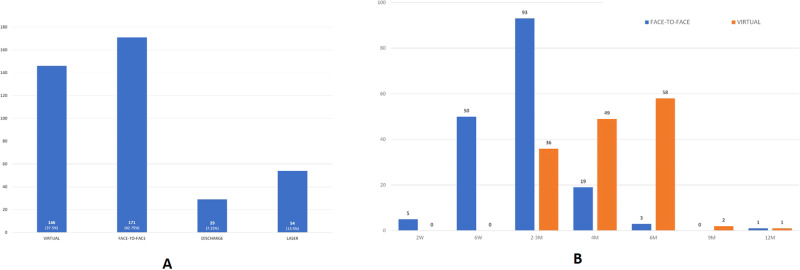
Diabetic Retinopathy Imaging Clinic (DRIC) Outcomes. Patient outcomes following assessment in the virtual DRIC (**A**) and subsequent follow up times in face to face and virtual clinic (**B**).

This study included 400 patients with a mean age 62.6 years, age range 20–92 years and male to female ratio of approximately 1.5:1. Outcomes and follow-up times for patients are presented in Fig. 1. Laser done was all for maculopathy. 1.25% of cases required urgent face-to-face appointments within 2 weeks (wet AMD and RVO). Median time to follow-up for DR was 2 months for face-to-face and 4 months for virtual appointments. 95.25% of patients were offered an initial appointment within the target of 13 weeks from referral from the DESP as set out by national guidelines [3].

Table 1 describes service provision for routine referrals before, during and after the COVID-19 pandemic. The number of referrals received, and appointments offered within the target 13-week timeframe reduced dramatically during Q1-Q2 2020 due to the pandemic. The introduction of the DRIC restored this to pre-pandemic levels.

**Table 1:** Number of routine DR referrals received before, during and after the introduction of the Diabetic Retinopathy Imaging Clinic (DRIC).

		Number of R2/M1 referrals	Offered within 13-week target	% Offered
**Pre-COVID-19 pandemic**	Average quarterly 2017	125	125	100%
	Average quarterly 2018	182	179	98%
	Average quarterly 2019	147	144	98%
**Pre-DRIC**	Q1 2020 (APR-JUN)	20	9	***50%***
	Q2 2020 (JUL-SEP)	89	12	***13%***
**Post- DRIC**	Q1 2021 (APR-JUN)	124	123	99%
	Q2 2021 (JUL-SEP)	114	114	100%
	Q3 2021 (OCT-DEC)	137	134	98%

R2 and M1 refer to the RxMx diabetic retinopathy grading system and indicate pre-proliferative retinopathy and maculopathy respectively. Q1/2/3 refers to quarters 1, 2 and 3 of the financial year, Q1 April to June, Q2 July to September, Q3 October to December inclusive.

## Discussion

The results of this study demonstrate that virtual review of new routine referrals from the DESP is safe and effective. It reduces need for face-to-face appointments and helps to meet national guidance of 13-week target for new referrals. To the authors knowledge, this is the first available data of a large cohort of new patients referred from DESP in the UK seen only virtually for the first visit and managed from virtual review. Other literature published to date demonstrates that virtual review is appropriate for a up to 74% of medical retina follow-up patients [4–6]. The virtual DRIC allowed for new patients to be seen more quickly and demonstrated that a significant proportion of new referrals from DESP are suitable for continued virtual follow-ups reducing waiting times, need for face-to-face appointments and enabling early treatment of sight threatening maculopathy. Future work should continue to assess the safety and acceptability of virtual clinics for diabetic retinopathy, particularly longer-term outcomes for patients managed entirely virtually.
