# Correction: Virtual front door for new referrals from a Diabetic Eye Screening Programme in England

**DOI:** 10.1038/s41433-023-02923-z

**Published:** 2024-01-11

**Authors:** Heather M. Wood, Sarita Jacob

**Affiliations:** 1 University Hospitals Birmingham NHS Trust, Birmingham, England; 2 College of Medical and Dental Sciences, University of Birmingham, Birmingham, England; 3 Birmingham, Solihull and Black Country Diabetic Eye Screening Programme, Birmingham, England; 4 College of Health and Life Sciences, Aston University, Birmingham, England

**Keywords:** Health services, Education

Correction to: *Eye* 10.1038/s41433-023-02865-6, published online 30 November 2023

The given names of the authors should be given in full as follows: ‘H. M. Wood’ has been corrected to ‘Heather M. Wood’ and ‘S. Jacob’ has been corrected to ‘Sarita Jacob’. The original article has been corrected.

